# Estimates of Paracetamol Poisoning in Brazil: Analysis of Official Records From 1990s to 2020

**DOI:** 10.3389/fphar.2022.829547

**Published:** 2022-03-08

**Authors:** Okuyama JHH, Galvao TF, Silva MT

**Affiliations:** ^1^ Graduate Program in Pharmaceutical Sciences, Universidade de Sorocaba, Sorocaba, Brazil; ^2^ School of Pharmaceutica Sciences, Universidade Estadual de Campinas, Campinas, Brazil

**Keywords:** admission, acetaminophen, suicide, paracetamol, poisoning

## Abstract

**Objective:** To assess the cases of paracetamol poisoning in Brazil. Methods: Analysis of official records of deaths between 1996 and 2019 from the Brazil Mortality Information System (SIM), admissions between 2008 and 2020 from the Hospital Information System (SIH), and cases of poisoning between 2017 and 2020 in health services, reported to the Brazilian Notifiable Diseases Surveillance System (SINAN). In SIM and SIH, records with ICD-10 were included: F55, T39, X40, X60, and Y10. In SINAN, commercial products containing paracetamol were identified. Records were stratified by age, sex, and intentionality. Mean and standard error were calculated for each stratum based on the annual data, by federation unit. Poisoning reports by 1,000,000 inhabitants were calculated from each state and compared to the national average. Results: In total, 492 deaths, 5,666 hospital admissions, and 17,031 cases of paracetamol poisoning were recorded in the period. Deaths occurred mostly among adults (71.3% ± 3.0) and in suicide attempts (37.3% ± 2.7). Hospital admissions were more frequent in adults (69.7% ± 1.4), women (57.1% ± 2.5), and unintentional poisoning (80.2% ± 4.2). Poisoning reports was more also frequent among adults (71.4% ± 1.2), women (74.2% ± 0.6), and due to accidents (79.6% ± 1.8). The South and Southeast regions of the country presented the highest frequencies in all outcomes, above the national average. Conclusion: Paracetamol exposure is a concern for preventable poisonings, hospital admissions and deaths. More accurate data about paracetamol poisoning are required to support surveillance actions and the development of mechanisms to reduce poisoning, particularly related to adults, women and suicide attempts.

## Introduction

Paracetamol is one of the most popular over-the-counter medicines worldwide. As a nonopioid analgesic and antipyretic agent, paracetamol is one of the best-selling drugs in Brazil (Brasil, 2019b). It is commercially available as tablets or oral solutions, isolated or associated with other active ingredients (Brasil, 2019a). It can be easily bought with or without a prescription, representing a common cause of accidental or intentional poison exposure (Park et al., 2015).

Reports of paracetamol poisoning are frequent and studied in several countries (Sheridan et al., 2017; Benlamkaddem et al., 2018; Parry et al., 2018). Drug abuse and self-medication for the treatment of chronic pain are associated with accidental poisoning (Ghanem et al., 2016). In cases of suicide attempts, the over-the-counter accessibility of paracetamol and high risk of hepatotoxicity, emergency interventions are frequently required (Stravitz and Lee, 2019).

Paracetamol is in the list of essential medicines and, in general, it is considered a safe drug (WHO, 2019b). However, acute liver injury is observed when used in doses above 10 g/day (Buckley et al., 1999) and, depending on nutritional or clinical conditions, lower doses can also cause hepatotoxicity (Kwan et al., 1995). The lowest acute dose capable of causing toxicity in adults is 7.5 g and in children 150 mg/kg (Bizovi and Smilkstein, 2002). In cases of paracetamol overdose, the adverse effect observed is acute liver failure, the main cause of fulminant hepatitis. The mechanism of hepatotoxicity is associated with the production of N-acetyl-*p*-benzoquinone imine, glutathione depletion and liver cell necrosis (Miner and Kissinger, 1979).

Warnings or educational campaigns highlighting the inadequate uses of paracetamol proposed by regulatory bodies have been conducted to reduce poisoning rates in the United States (FDA, 2017). Although the risks of paracetamol are well known, estimates of paracetamol poisoning have been poorly investigated in Brazil. The risk of this exposure does not seem to be lower in the country, as the Brazilian National Health Surveillance Agency (ANVISA) warned in 2021 to the risk of drug-induced hepatitis after prolonged use or drug abuse in situations of indiscriminate use of paracetamol to relieve the adverse events of vaccines against COVID-19 (Brasil, 2021).

This study aimed to identify and analyze paracetamol poisoning in Brazil.

## Methods

### Study Design

This is a descriptive retrospective study that used different information systems to obtain representative data about paracetamol poisoning in Brazil. Three database systems were assessed: the Brazilian Mortality Information System (SIM), the Hospital Information System (SIH), and the Brazilian Notifiable Diseases Surveillance System (SINAN). Table 1 summarizes the data collected from these three information systems.

**TABLE 1:** Characteristics and strategies for the identification of paracetamol poisoning information in official databases.

	Mortality Information System	Hospital Information System	Brazilian Notifiable Diseases Surveillance System
Study period	1996–2019	2008–2020	2017–2020
Origin of collected data	SUS IT Department (DATASUS)—SIM	DATASUS—SIH	DATASUS—SINAN
Website	https://datasus.saude.gov.br/mortalidade-desde-1996-pela-cid-10	https://datasus.saude.gov.br/acesso-a-informacao/producao-hospitalar-sih-sus/	https://datasus.saude.gov.br/acesso-a-informacao/doencas-e-agravos-de-notificacao-de-2007-em-diante-sinan/
National representativeness	Yes	Restricted to SUS	Yes
Estimates	All paracetamol-related deaths	All SUS paracetamol-related admissions	All paracetamol poisoning cases treated in health services
Definitions adopted	Data processed according to ICD-10: F55^1^; T39^2^; X40^3^; X60^4^; Y10^5^	Data processed according to ICD-10: F55^1^; T39^2^; X40^3^; X60^4^; Y10^5^	Circumstance of exposure/contamination: 02 (accidental); 05 (inadequate prescription); 06 (administration error); 07 (self-medication); 08 (abuse); 10 (suicide attempt); 12 (violence/homicide); 13 (other); 99 (ignored)
Causes	**Suicide:** X60^4^ (ICD-10)	**Suicide:** X60^4^ (ICD-10)	**Suicide:** 10 (suicide attempt)
	**Unintentional:** F55^1^; T39^2^; X40^3^; Y10^5^ (ICD-10)	**Unintentional:** F55^1^; T39^2^; X40^3^; Y10^5^ (ICD-10)	**Unintentional:** 02 (accidental); 05 (inadequate prescription); 06 (administration error); 07 (self-medication); 08 (abuse); 12 (violence/homicide)
	—	—	**Ignored/Other:** 13 (other); 99 (ignored)

Notes: ^1^Abuse of non-dependence-producing substances; ^2^Poisoning by nonopioid analgesics, antipyretics and antirheumatics; ^3^Accidental poisoning by and exposure to nonopioid analgesics, antipyretics and antirheumatics; ^4^Intentional self-poisoning by and exposure to nonopioid analgesics, antipyretics and antirheumatics; ^5^Poisoning by and exposure to nonopioid analgesics, antipyretics and antirheumatics, undetermined intent.

### Background

SIM was created by the Informatics Department of the Unified Health System (DATASUS) and implemented in Brazil in 1975 by the Ministry of Health. The system combines statistical information from death forms recorded at the municipal, state and federal levels of public health management. The death form is standardized, filled in by the physician and collected by the Municipal or State Health Department. It is a free file transfer protocol (FTP) system, and allows users to view files and join files from different periods as a historical series, combining them into the same file.

SIH processes hospital admission records from the Unified Health System (SUS) patients in Brazil. Each record has a Hospital Admission Authorization, allowing the payment of fixed amounts for hospital and medical procedures by the SUS. This system displays municipal and state data obtained from the Ministry of Health website by free FTP system.

SINAN receives data from disease investigations of compulsory reporting; its updating is mandatory at the municipal, state, and federal levels. Poisoning cases treated at health services involve compulsory reporting in Brazil, which must be made in SINAN system. Consolidated data can be freely accessed via DATASUS. The poisoning agent insertion in the system happens by free text and up to three agents under the trade name and three active ingredients can be provided, which may involve spelling mistakes.

### Participants

This study assessed the cases of death from paracetamol poisoning between 1996 and 2019 from SIM under the codes of the 10th revision of the International Classification of Diseases (ICD-10) as primary and secondary causes and probable circumstances of unnatural death, as follows: F55 (abuse of non-dependence-producing substances), T39 (poisoning by nonopioid analgesics, antipyretics and antirheumatics), X40 (accidental poisoning by and exposure to nonopioid analgesics, antipyretics and antirheumatics), X60 (intentional self-poisoning by and exposure to nonopioid analgesics, antipyretics and antirheumatics), and Y10 (poisoning by and exposure to nonopioid analgesics, antipyretics and antirheumatics, undetermined intent); SUS admissions from 2008 to 2020 with primary and secondary diagnosis registered in SIH as F55, T39, X40, X60, and Y10, according to ICD-10; and cases of paracetamol poisoning between 2017 and 2020 reporting it as the toxic agent. The period defined was feasible considering data availability in FTP for each database system.

### Variables

The primary outcomes were death (1996–2019), SUS admission (2008–2020), and poisonings treated and reported in health services (2017–2020). The independent variables were age (in years: <6, 6-16, 17-64, ≥65) and sex (male, female). For a better understanding of the poisoning causes, they were classified as “unintentional” (accidental, inadequate drug prescription, medication administration error, self-medication, drug abuse, and violence or homicide), “suicide attempt,” and “not informed/ignored.” The variable “state of residence” was also assessed in SINAN poisoning reports.

### Data Source and Measurement

Data were obtained from the repositories and compiled in a Microsoft Excel^®^ spreadsheet.

Because the toxic agent information is manually inserted in the SINAN form, paracetamol poisoning was identified by searching all names and spelling variations for paracetamol. We created a list of commercial presentations with paracetamol based on an inquiry in active and discontinued drug registries on the ANVISA website (https://www.gov.br/anvisa/pt-br/acessoainformacao/dadosabertos/informacoes-analiticas) for this purpose. This inquiry was performed in September 2019 in both expired drug registrations and registrations in force.

The reports of poisoning cases in the SINAN were grouped by state and calculated by the respective population official estimate according to the Brazilian Institute of Geography and Statistics (http://ibge.gov.br). The rate of poisonings per million inhabitants for each state (federation unit) was calculated for 2017 to 2020.

### Statistical Analysis

We used the SIM, SIH and SINAN databases to address different hypothesis about paracetamol poisoning. We investigated whether there is a difference in terms of age, sex and causes for each outcome. For each stratum, we calculated the percentage for each group by year and calculate the mean and standard error of these percentages over each assessed year. We performed two-tailed analysis of variance (ANOVA) for independent samples to detect any difference in each stratum and considered an alpha level of 0.05 to assign statistical significance. The rate of poisoning per 1 million inhabitants was described by federation unit and compared to the national average. The state rate was also plotted in map. All analyzes were performed in Microsoft Excel^®^.

## Results

A total of 492 deaths from paracetamol poisoning were identified in Brazil between 1996 and 2019, of which 183 were cases of suicide and 123 were accidental deaths (*p* < 0.004). Deaths were more frequent in the population over 17 years of age (*p* < 0.001, Table 2).

**TABLE 2:** Summary of paracetamol poisoning data obtained from different official records.

Variables	Mortality (1996–2019)		Admissions (2008–2020)		Poisoning reports (2017–2020)	
	Mean ± SE	*p*-value	Mean ± SE	*p*-value	Mean ± SE	*p*-value
Age (years)	—	<0.001*	—	<0.001*	—	<0.001*
<6	7.3 ± 1.6	—	12.6 ± 0.9	—	6.2 ± 0.8	—
6–16	5.1 ± 1.2	—	13.4 ± 0.9	—	21.5 ± 0.6	—
17–64	71.1 ± 3.0	—	69.7 ± 1.4	—	71.4 ± 1.2	—
≥65	16.6 ± 2.0	—	4.4 ± 0.4	—	0.9 ± 0.1	—
Sex	—	0.611**	—	<0.001**	—	<0.001**
Male	50.9 ± 2.5	—	42.9 ± 2.5	—	25.8 ± 0.6	—
Female	49.1 ± 2.5	—	57.1 ± 2.5	—	74.2 ± 0.6	—
Cause of exposure	—	<0.004***	—	<0.001***	—	<0.001***
Suicide	37.3 ± 2.7	—	19.8 ± 4.2	—	20.4 ± 1.8	—
Unintentional	25.7 ± 3.5	—	80.2 ± 4.2	—	79.6 ± 1.8	—
Not reported/ignored/other	37.0 ± 3.1	—	—	—	—	—

*p value from ANOVA, for age; **p value from ANOVA, for sex; and ***p value from ANOVA, for cause.

From 2008 to 2020, 5,666 admissions to due to paracetamol poisoning occurred in SUS hospitals. Women, adults and unintentional reason were the most affected stratum (*p* < 0.001, Table 2). Intentional use of paracetamol in suicide attempts represented 19.8% of admission in Brazilian public hospitals (*p* < 0.001, Table 2).

From 2017 to 2020, 515,539 poisoning cases were reported by Brazilian health services, of which 17,031 (3.3%) were paracetamol poisonings. Women and people aged between 17 and 64 years (*p* < 0.001) were more frequently seen in poisoning cases (Table 2). Suicide attempts accounted 20.4% of the reports (*p* < 0.001).

**FIGURE 1:**
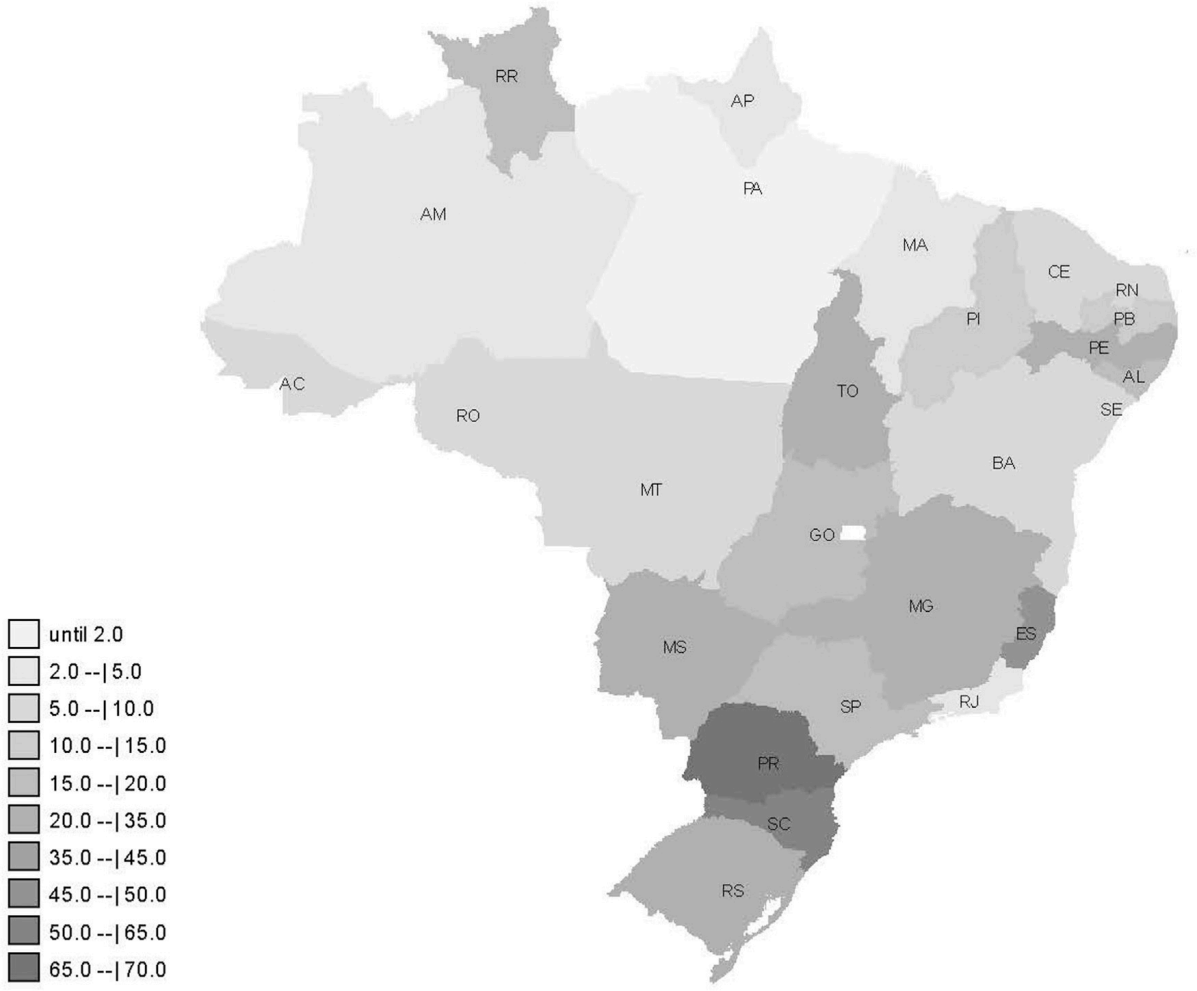
Distribution of paracetamol poisoning cases per million inhabitants reported from 2017 to 2020 in Brazil.

In the whole country, it was reported 20.3 cases of paracetamol poisoning per million inhabitants in this period. Paracetamol poisoning reports were concentrated in the South and Southeast regions (Figure 1). The state of Espírito Santo (ES), Southeast Region, had 2.3 times more cases than the national average (45.8 per million). In the South Region, the state of Paraná (PR) had 3.3 times more (67.2 per million) and Santa Catarina (SC), had 3.0 times more (61.6 per million) than the national average.

## Discussion

In Brazil, paracetamol exposures leading to poisonings and hospital admissions were higher among women and adults. When compared to unintentional poisoning, deaths from paracetamol exposures due to suicide were significantly higher. This research represents the first effort to bring an overall assessment of the dimension of paracetamol exposure in Brazil.

Despite the completeness of present investigation, our results are limited by the quality of the records. Although the mortality system (SIM) includes all deaths in Brazil, we suspect that there is underreporting. The ICD-10 included probably refers to acute exposures and/or more severe cases, with better investigations. It is also possible that suicides are underestimated, due to life insurance and inheritance issues. Only hospitalizations funded by the SUS were included. Hospitalizations paid for by the patient or by private health insurance could not be included. Brazil has 270,880 general beds, of which 66% are available for the SUS (Noronha et al., 2020). On the other hand, the first urgent and emergency care, common in cases of intoxication, is virtually covered only by the SUS. Reports of poisoning treated in Brazilian health services—public or private—are mandatory and would include more serious cases requiring medical care, and lack representativeness of all poisoning cases. Non-standardization of the SINAN form in its toxic agent information field may lead to failed records and possible information bias. The retrospective design based on administrative databases also impacts the reliability of the findings. Poisoning data identification and analysis in official databases, similar to present effort, are widely used as a surveillance method in other countries (Nourjah et al., 2006; Gedeborg et al., 2017) and are considered valuable sources to assess the magnitude of the problem.

Deaths, hospital admissions, and poisoning reports due to paracetamol exposure were predominant among adults, a scenario that is similar to what was observed in Sweden in 2000–2013 (Gedeborg et al., 2017), Germany in 2007–2018 (Daly et al., 2020), England in 2004–2014 (Casey et al., 2020), and Algeria in 2010–2017 (restricted to one hospital) (Chefirat et al., 2020).

Paracetamol administration above the maximum recommended dose of 4 g/day and chronic use for pain control are the main causes of poisoning among adults in the United States (Blieden et al., 2014). Therapeutic effects of paracetamol such as analgesia and antipyretics are considered safe and accepted (Bertolini et al., 2006), which makes it possible to use it in a wide age range, from young children to elderly people, to control fever and pain (WHO, 2019b), preferable to non-steroidal anti-inflammatory drugs (NSAIDs), to avoid gastric irritation. It was considered a first choice drug for pain control by the American Pain Society in conjunction with the American College of Physicians along with NSAIDs in pain control (Chou et al., 2007). Easy access, no prescription required, and self-medication favor poisoning. The use of paracetamol to control pain in osteoarthritis is known and often indicated due to its safety when compared to NSAIDs (Shah and Mehta, 2012). Chronic pain management in these conditions should use a combination of pharmacological and non-pharmacological treatments (physical and occupational therapies, behavioral and cognitive therapies, nutritional therapy, among others) (Borenstein et al., 2017), which can help patients live with chronic pain and avoid opioid dependence or drug poisoning (Kjartansdottir et al., 2012; Conaghan et al., 2019).

Regulatory agencies in settings with high rates of suicide attempts and suicide with paracetamol propose alternatives such as limitations to the amount sold over-the-counter, restricting sales to medical prescription, and changes in commercial packaging to mitigate the risks (Gunnell et al., 2000). In Brazil, drug combinations of paracetamol with opioid analgesics has special control, such as the compounds of paracetamol associated with tramadol and codeine (Brasil, 1998). During the opioid overdose crisis, the United States Food and Drug Administration decreased the concentration of paracetamol in the commercial presentation of paracetamol and hydrocodone after a public consultation to reduce cases of paracetamol poisoning (Blieden et al., 2014).

Hospital admissions caused by paracetamol poisoning were more frequent among women in Brazil. In a multicenter study conducted in England from 2011 to 2014, women presented higher rates of paracetamol poisoning than men (Casey et al., 2020). Men had a higher death rate despite lower admissions than women due to higher doses of paracetamol. Mental health problems such as depression are more frequent in women (WHO, 2013) than men. Factors such as social context, failure to seek help for mental health issues, and skills and access to weapons between men and women (Möller-Leimkühler, 2003) explain the double rate of consummated suicide in men (WHO, 2019a), while the frequency of suicide attempts is higher in women (Canetto and Sakinofsky, 1998). Psychological and social factors and cultural characteristics also influence the means used in suicide attempts by men and women, which are more lethal in men (Varnik et al., 2011; Mergl et al., 2015).

Hospital admissions due to unintentional poisoning were more frequent than intentional exposure to paracetamol. On the other hand, when analyzing deaths, suicide attempts were more frequent. The indiscriminate use of medication to control pain increases unintentional poisoning, with simultaneous administration of two or more formulations with paracetamol (Larson et al., 2005). Poor knowledge of consumers about adverse events related to the drug may also increase risks and unintentional poisoning (Daifallah et al., 2021). In addition to the consumption of different compounds with acetaminophen, easy access without a prescription, and self-medication, the concomitant use with alcohol further damages the liver function and increases the risk of unintentional poisoning (Kane et al., 2012; Kjartansdottir et al., 2012; Hawton et al., 2019). In 2016, unintentional poisoning using different toxic agents caused 100,000 deaths worldwide (WHO, 2019c) and medicines were the main cause of admissions, mainly among adults (Yehya et al., 2020).

Paracetamol poisoning in Brazil was more concentrated in the South and Southeast regions. These regions have higher Human Development Index and per capita income (Organização das Nações Unidas, 2021). They also have more Poison Control Centers. These aspects culturally influence the number of reports in health services, recognition the importance of this condition and its mandatory reporting. In the American context, population characteristics–such as ethnicity, race, educational level, population with the largest number of children, and per capita income–influenced the number of toxic exposure reports in the Poison Control Centers (Litovitz et al., 2010). Differences in the accessibility and structure of services can also affect reporting rates and types (Laborde, 2004). Unlike the United States, Brazil has a public healthcare system, accessible to all Brazilians, despite its budgetary underfunding.

Given the consumption of paracetamol in the Brazilian population, further studies on paracetamol poisoning could assess data from all Poison Control Centers in Brazil such as performed for pesticides in 2017 (Okuyama et al., 2020) and the association of paracetamol with other drugs, such as codeine and tramadol, which are used to control moderate pain.

## Conclusion

The results found in this study indicate that cases of paracetamol exposure are a reality in the Brazilian scenario and exposure are a concern for avoidable poisoning, hospital admission and deaths. The adult population was more affected by paracetamol poisoning leading to death, and was also the most frequent group in SUS admissions and healthcare services due to paracetamol poisoning in Brazil. Women and unintentional causes were more common in public hospital admissions and in poisonings treated in Brazilian healthcare services. Deaths were higher due to suicide. Estimates of paracetamol poisoning should be based on more accurate data that allows surveillance, monitoring and mechanisms to avoid cases of chronic exposure and suicide.

## Data Availability

The original contributions presented in the study are included in the article/Supplementary Material, further inquiries can be directed to the corresponding author.
